# Long non-coding RNA MUC5B-AS1 promotes metastasis through mutually regulating MUC5B expression in lung adenocarcinoma

**DOI:** 10.1038/s41419-018-0472-6

**Published:** 2018-04-18

**Authors:** Shuai Yuan, Qingyun Liu, Zeyao Hu, Zhengyu Zhou, Guilu Wang, Chengying Li, Weijia Xie, Gang Meng, Ying Xiang, Na Wu, Long Wu, Zubin Yu, Li Bai, Yafei Li

**Affiliations:** 1 Department of Epidemiology, College of Preventive Medicine, Army Medical University (Third Military Medical University), 400038 Chongqing, China; 2 Department of Pathology, Southwest Hospital, Army Medical University (Third Military Medical University), 400038 Chongqing, China; 3 Department of Thoracic Surgery, Xinqiao Hospital, Army Medical University (Third Military Medical University), 400038 Chongqing, China; 4 Department of Respiratory Disease, Xinqiao Hospital, Army Medical University (Third Military Medical University), 400038 Chongqing, China

## Abstract

Long non-coding RNAs (lncRNAs) have been involved in the process of cancer occurrence, progression, and treatment. Lung cancer-related lncRNAs are still an emerging field, thus we sought to identify novel functional lncRNAs as candidate targets in lung cancer. Here, we identified one novel lncRNA, MUC5B-AS1 (Ensembl: ENST00000532061.2). MUC5B-AS1 was upregulated in lung adenocarcinoma tissues compared with normal lung tissues. Moreover, MUC5B-AS1 promoted lung cancer cell migration and invasion *in vitro* and promoted lung cancer cell metastasis *in vivo*. MUC5B-AS1 and its cognate sense transcript MUC5B were highly co-expressed and mutually regulated in lung adenocarcinoma. Mechanistically, MUC5B-AS1 promoted cell migration and invasion by forming an RNA–RNA duplex with MUC5B, thereby increasing MUC5B expression levels in lung adenocarcinoma. The high expression of MUC5B was significantly associated with poor outcomes in lung adenocarcinoma. Our findings highlight MUC5B-AS1 functions as an oncogenic lncRNA in tumor metastasis and implicate MUC5B-AS1 as an attractive candidate target for lung adenocarcinoma treatment.

## Introduction

Lung cancer continues to be the most common malignancies and the leading cause of cancer mortality worldwide^1,2^. Adenocarcinoma is the major subtype of lung cancer^3^. Although the discovery of oncogenes and tumor suppressor genes leads to profound insights into the mechanisms of lung cancer, the overall 5-year survival rate is less than 20%^3^. The poor prognosis of lung cancer is largely due to early metastasis^4^. Thus, identification of novel therapeutic targets for invasion and metastasis may provide alternative approaches for management of patients with lung cancer.

Long non-coding RNAs (lncRNAs) are a class of functional transcripts longer than 200 nucleotides with no protein-coding capacity^5^. Accumulating evidences have suggested that majority of lncRNAs are likely to regulate the targeted gene transcription^6^, and also function in post-transcriptional^7^ and epigenetic regulation of genes^8^. LncRNAs have been also involved in the process of cancer occurrence, progression and treatment, such as breast cancer^9^, colorectal cancer^10^, and hepatocellular carcinoma^11^. Lung cancer-related lncRNAs are still an emerging field, thus we sought to identify novel functional lncRNAs as candidate targets in lung cancer.

We have screened differentially expressed lncRNAs in five paired lung cancer tissues using Affymetrix GeneChip Human Transcriptome Array 2.0, which identified 332 lncRNAs significantly differentially expressed between tumor and paired normal lung tissues. The quantitative real-time PCR (qRT-PCR) analysis confirmed one lncRNA, MUC5B-AS1 (Ensembl: ENST00000532061.2) was significantly upregulated in lung adenocarcinoma. MUC5B-AS1 is a novel long non-coding antisense transcript for MUC5B.

MUC5B is a member of the mucin (MUC) family, which are highly glycosylated macromolecular components of mucus secretions. The human mucin family are structurally sub-classified into secreted and transmembrane forms, and MUC5B belongs to the secreted forms^12^. MUC5B is the dominant macromolecules in airway mucus and is required for airway defense in the healthy lung^13^. Variants of MUC5B is associated with the susceptibility of bladder cancer^14^, and the expression levels of MUC5B are upregulated in colorectal cancer^15^, gastric carcinoma^16^, and breast cancer^17^. Functional studies of MUC5B also revealed that MUC5B could promote breast cancer MCF7 cells proliferation and metastasis^18^. In lung cancer, previous studies have explored the relationships between MUC5B expression and clinicopathological characteristics, which found that overexpression of MUC5B was associated with early post-operative metastasis and poor overall survival (OS) in patients with lung adenocarcinoma^19–22^. However, whether lncRNA MUC5B-AS1 regulates MUC5B expression is unknown.

In the present study, we focus on the role and molecular mechanism of MUC5B-AS1 in lung cancer. We demonstrate that MUC5B-AS1 functions as an oncogenic lncRNA in lung cancer. Moreover, MUC5B-AS1 regulates the MUC5B expression through increasing the stability of MUC5B mRNA by forming a protective RNA–RNA duplex. We also evaluate the clinical implication of MUC5B-AS1 and MUC5B in lung cancer prognosis.

## Results

### Characterization and expression pattern of lncRNA MUC5B-AS1

**Fig. 1:**
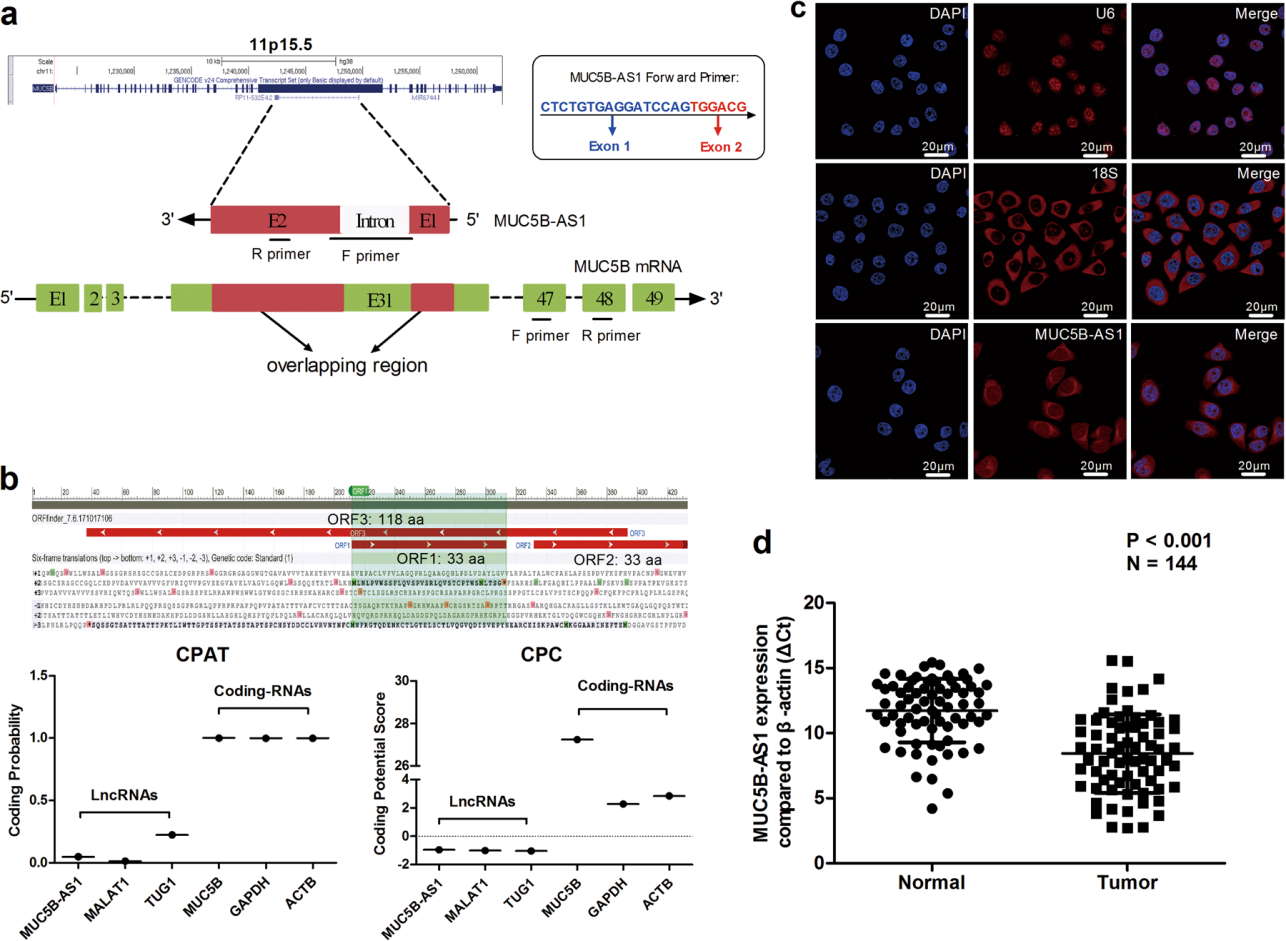
Identification of an overlapping antisense lncRNA at the MUC5B gene locus. **a** The localizations of MUC5B-AS1 and MUC5B on the UCSC genome browser. The schema was not drawn to scale. MUC5B-AS1 is located at chromosomal 11p15.5, and composes of two exons. MUC5B-AS1 is an antisense lncRNA, embedded on the opposite DNA strand of the MUC5B gene within its 31st exon. Green blocks indicate exons, and red blocks are overlapping regions. Primers of MUC5B-AS1 and MUC5B are also indicated in the schema: F primer forward primer, R primer reverse primer. The forward primer of MUC5B-AS1 spans the exon1–exon2 junction to avoid the non-specific amplification of MUC5B mRNA or genomic DNA. **b** Upper chart: ORF prediction of MUC5B-AS1 sequence. Three potential ORFs that might code peptides of 33–118 amino acids are present in the MUC5B-AS1 sequence. Lower chart: coding potentials of lncRNAs (MUC5B-AS1, MALAT1, TUG1) and mRNA (MUC5B, GAPDH, ACTB) were calculated using CPAT and CPC. **c** Localization of MUC5B-AS1 by RNA-FISH. Blue, DAPI-stained nuclei; red, Cy3-labeled positive hybridization signals (scale bar, 20 μm). The U6 and 18S were used as positive control. **d** Expression analysis of MUC5B-AS1 in lung adenocarcinoma tissues (*n* = 72) and paired adjacent normal lung tissues (*n* = 72). The ΔCt was used to show the expression level of MUC5B-AS1 (ΔCt = Ct_MUC5B-AS1_–Ct_*β-actin*_). Lower ΔCt values indicate higher expression. Normal vs. tumor tissues, Student’s *t*-test

According to the UCSC genome browser, ENST00000532061.2 is identified as a single-strand antisense lncRNA that is transcribed from the negative strand of the MUC5B locus, thus we named it MUC5B-AS1. MUC5B-AS1 is located at chromosomal 11p15.5, and composed of two exons with a full length of 434 nt (Fig. 1a, Supplementary Fig. S1a). The 31st exon of MUC5B shares a 434-nucleotide-long region with full length of MUC5B-AS1 (Fig. 1a). We referred to this region as the overlapping (OL) region. Specific primers were designed to amplify MUC5B-AS1 and MUC5B mRNA. The forward primer of MUC5B-AS1 spans an exon–exon junction to avoid the non-specific amplification of MUC5B mRNA or genomic DNA (Fig. 1a, Supplementary Fig. S1b). Using the Open Reading Frame (ORF) Finder (https://www.ncbi.nlm.nih.gov/orffinder/), we analyzed the sequence of MUC5B-AS1 and identified three potential ORFs that might code peptides of 33–118 amino acids (Fig. 1b). In order to confirm the non-coding nature of MUC5B-AS1, coding-potential analysis was performed by Coding Potential Assessment Tool (CPAT)^23^ and Coding Potential Calculator (CPC) computational algorithm^24^, which predicted that MUC5B-AS1 had a very low coding potential (Fig. 1b). We also performed an RNA fluorescence in situ hybridization (FISH) assay to examine the subcellular localization of MUC5B-AS1. The data revealed that MUC5B-AS1 was localized both in the nucleus and cytoplasm and predominantly in the cell cytoplasm (Fig. 1c). We further dissected the subcellular localization of MUC5B mRNA. Consistent with the results of MUC5B-AS1, MUC5B mRNA was also predominantly observed in the cell cytoplasm (Supplementary Fig. S1c). Next, we explored MUC5B-AS1 expression pattern in 72 paired lung adenocarcinoma tumor and adjacent normal lung tissues using qRT-PCR. MUC5B-AS1 expression was significantly upregulated in lung cancer tissues compared with adjacent normal tissues (*P* < 0.001) (Fig. 1d). MUC5B-AS1 expression levels were associated with TNM stage. Patients with advanced TNM stage (II/III/IV, *n* = 34) showed higher MUC5B-AS1 expression levels than patients with early TNM stage (I, *n* = 38) (9.019 ± 0.499 vs. 7.790 ± 0.487, *P* = 0.084) (Supplementary Fig. S1d).

### MUC5B-AS1 overexpression promotes lung cancer cell migration and invasion *in vitro*

**Fig. 2:**
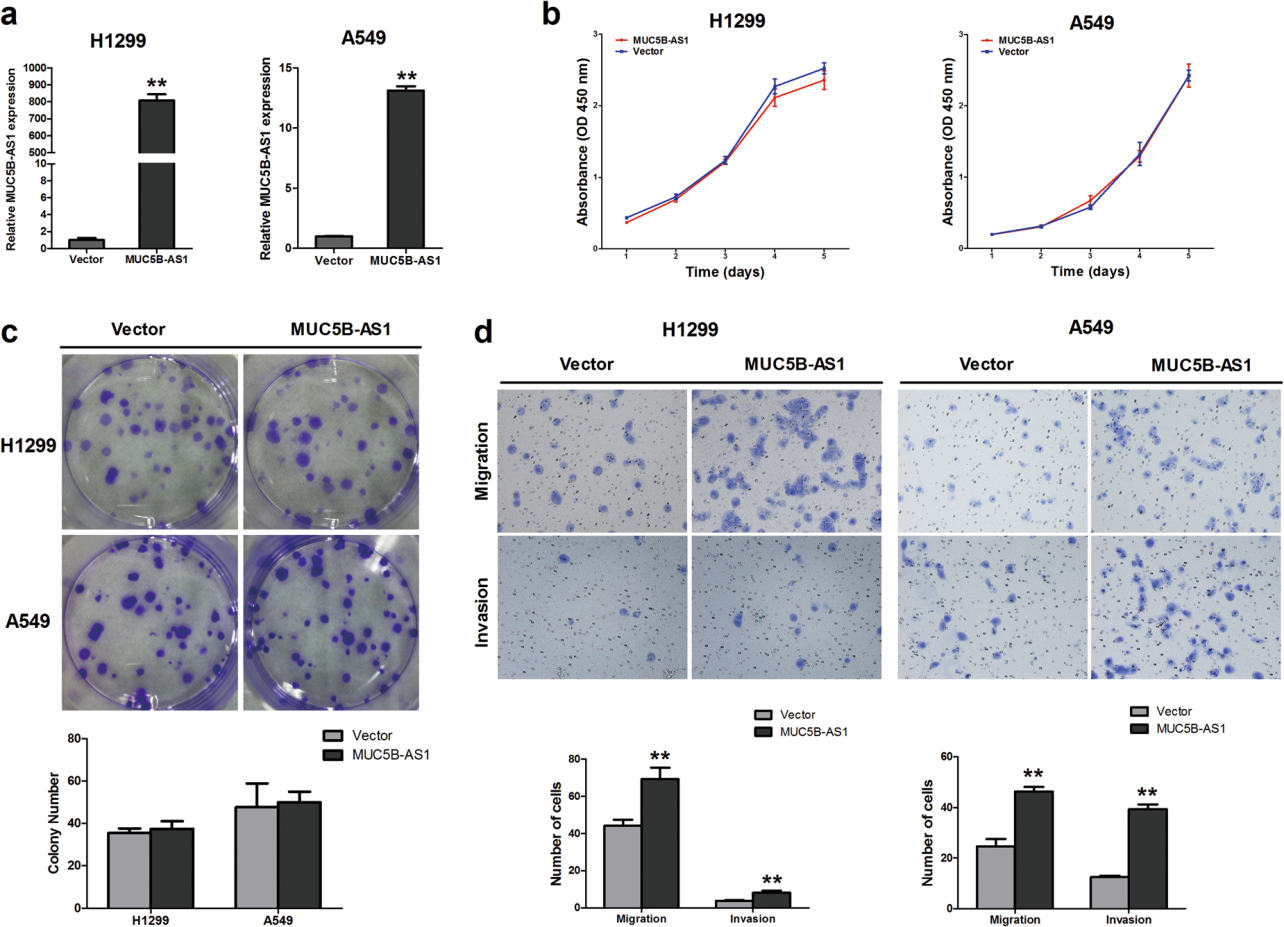
Effect of MUC5B-AS1 overexpression on cell proliferation, migration, and invasion. **a** Overexpression of exogenous MUC5B-AS1 in H1299 and A549 cells were identified by qRT-PCR. **b** CCK-8 assays were used to examine the effect of MUC5B-AS1-overexpressing on proliferation in H1299 and A549 cells. **c** The effect of MUC5B-AS1 on cell growth was further detected by a colony formation assay. Visible colonies were counted. **d** Transwell assays were used to investigate the effect of MUC5B-AS1 on migration and invasion in H1299 and A549 cells. Error bars represent the SD of three independent experiments. MUC5B-AS1 vs. control vector, Student’s *t*-test, **P < 0.01

To explore the biological functions of lncRNA MUC5B-AS1 in lung adenocarcinoma, we established gain-of-function cell models by transfecting pcDNA3.1-MUC5B-AS1 expressing vectors into H1299 and A549 cell lines. The overexpression of exogenous MUC5B-AS1 was confirmed by qRT-PCR (Fig. 2a). We examined the effects of MUC5B-AS1 overexpression on cell proliferation, migration, and invasion. CCK-8 and colony formation assays showed that MUC5B-AS1 overexpression had no effect on the proliferation of H1299 and A549 cells (*P* > 0.05) (Fig. 2b, c). The transwell assays showed that overexpression of MUC5B-AS1 significantly increased the migration and invasion of H1299 and A549 cells (*P* < 0.01) (Fig. 2d).

### Knockdown of MUC5B-AS1 inhibits lung cancer cell migration and invasion *in vitro*

**Fig. 3:**
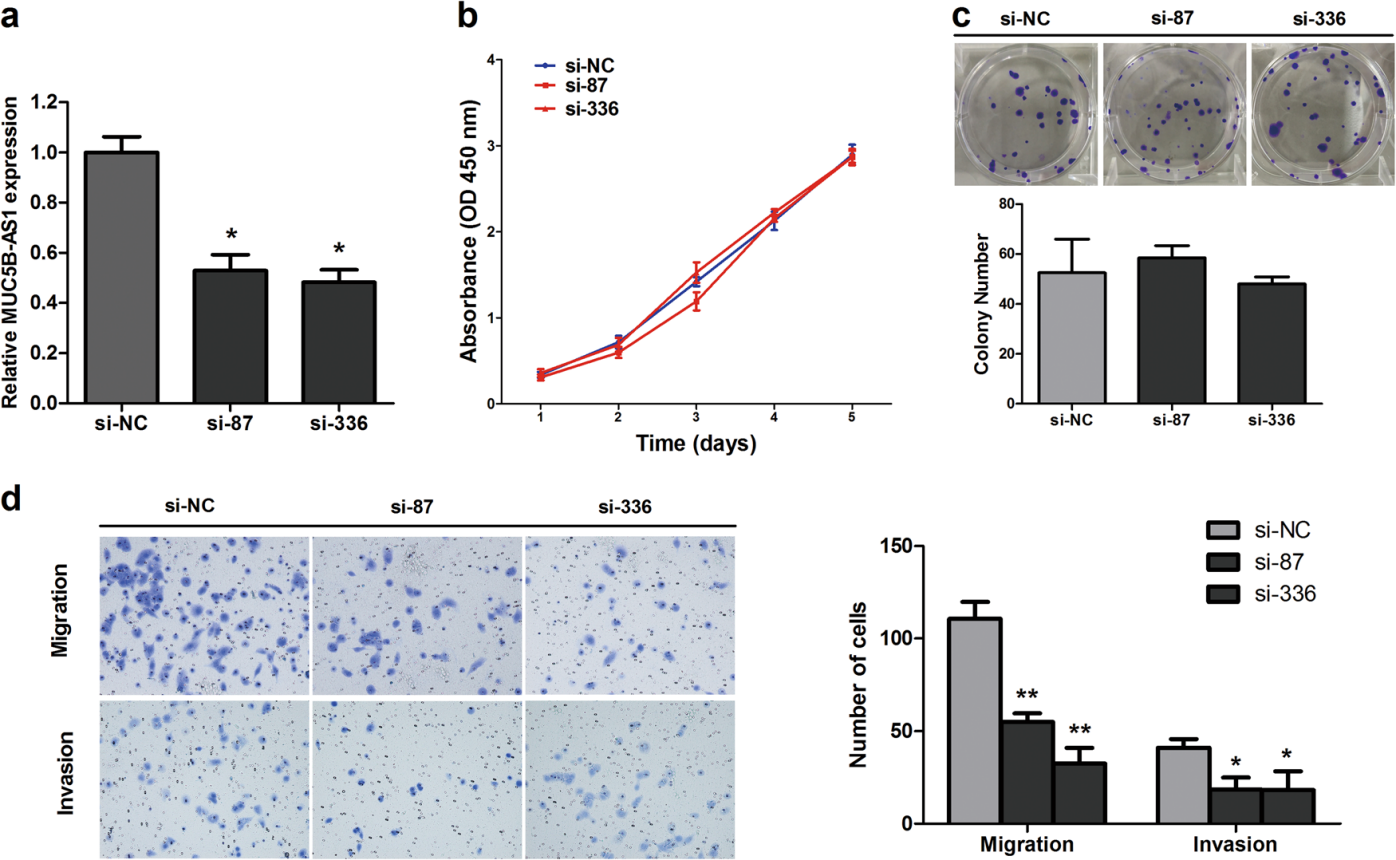
Effects of MUC5B-AS1 knockdown by siRNA on cell proliferation, migration, and invasion. **a** The knockdown expression of MUC5B-AS1 in A549 cells were identified by qRT-PCR. **b** CCK-8 assays were used to examine the effect of MUC5B-AS1 knockdown on proliferation in A549 cells. **c** The effect of MUC5B-AS1 knockdown on cell growth were also detected by a colony formation assay. Visible colonies were counted. **d** Transwell assays were used to examine the effect of MUC5B-AS1 knockdown on migration and invasion in A549 cells. Error bars represent the SD of three independent experiments. siRNA vs. si-NC, Student’s *t*-test, *P < 0.05. siRNA vs. si-NC, Student’s *t*-test, **P < 0.01

Next, we knocked down the expression of MUC5B-AS1 in A549 cells using small interfering RNAs (siRNAs). Due to the nucleotide base complementary relationship between MUC5B-AS1 and MUC5B, we designed two independent single-strand siRNAs targeting MUC5B-AS1 to avoid the interference of MUC5B (Supplementary Table S2). MUC5B-AS1 expression levels were significantly reduced after siRNAs transfection in A549 cells (Fig. 3a). Similar to the gain-of-function cell models, knockdown of MUC5B-AS1 had no effect on A549 cells proliferation (Fig. 3b) and clonogenic survival (Fig. 3c). However, the *in vitro* transwell assays showed that MUC5B-AS1 knockdown significantly impeded the migration and invasion of A549 cells (Fig. 3d).

### MUC5B-AS1 promotes lung cancer cell metastasis *in vivo*

**Fig. 4:**
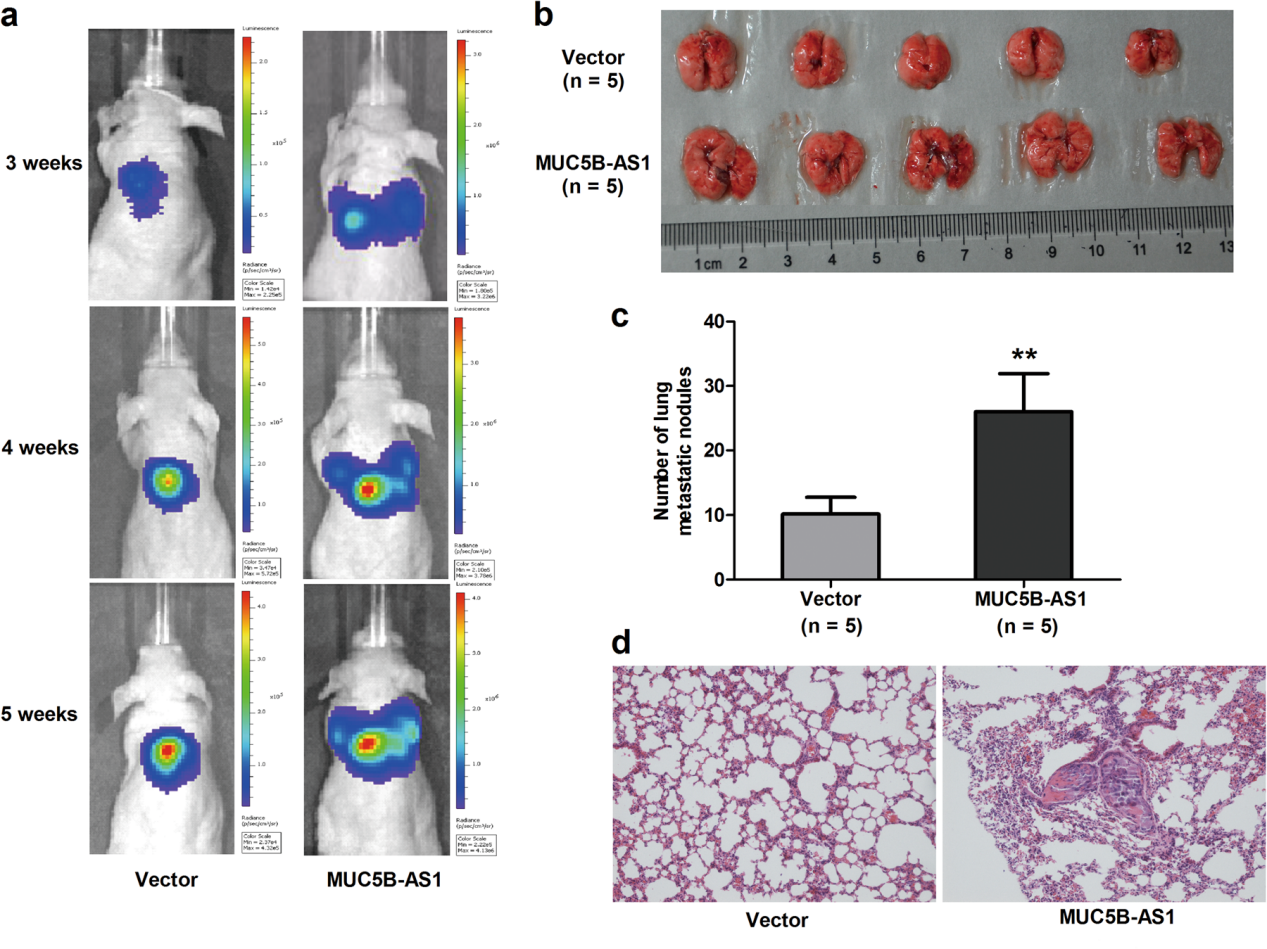
Overexpression of MUC5B-AS1 promotes lung cancer cell metastasis *in vivo*. **a** Representative bioluminescent images showed lung metastasis at 3, 4, 5 weeks post injection in nude mice. The mice were injected with MUC5B-AS1 stable overexpressing H1299 cells (firefly luciferase-labeled, 1 × 10^6^ cells per mice) via the lateral tail veins. Optical *in vivo* imaging of cancer metastasis was monitored with IVIS. **b** Photograph of entire lungs from nude mice in each group 5 weeks after injections of H1299 cells. **c** The number of lung metastatic nodules on lung surfaces were counted. MUC5B-AS1 vs. control vector, Student’s *t*-test, **P < 0.01. **d** Representative H&E staining of lung tissue slices confirmed that more metastatic nodules were present in MUC5B-AS1 group than vector control group (100×)

To further confirm the pro-metastatic potential of MUC5B-AS1 in lung cancer, we investigated whether MUC5B-AS1 could promote tumor metastasis *in vivo* using tail vein injection assays. *In vivo* optical imaging demonstrated that upregulation of MUC5B-AS1 increased lung colonization of H1299 cells at 3, 4, and 5 weeks post injection (Fig. 4a). The number of lung metastatic nodules was significantly increased in mice receiving MUC5B-AS1 stable overexpressing H1299 cells compared with those receiving cells transfected with vector control (*P* < 0.01) (Fig. 4b, c). The difference was further confirmed by the examination of the lungs through hematoxylin and eosin (H&E) staining of the mice lung sections (Fig. 4d). We confirmed the pro-metastatic potential of MUC5B-AS1 using A549 cells. The number of metastatic nodules on the surface of the lung was significantly increased in mice receiving MUC5B-AS1 stable overexpressing A549 cells compared with control (Supplementary Fig. S2a, b). The metastatic nodules were further confirmed by H&E staining of the mice lung sections (Supplementary Fig. S2c).

### MUC5B-AS1 and MUC5B are co-expressed and mutually regulated in lung adenocarcinoma

**Fig. 5:**
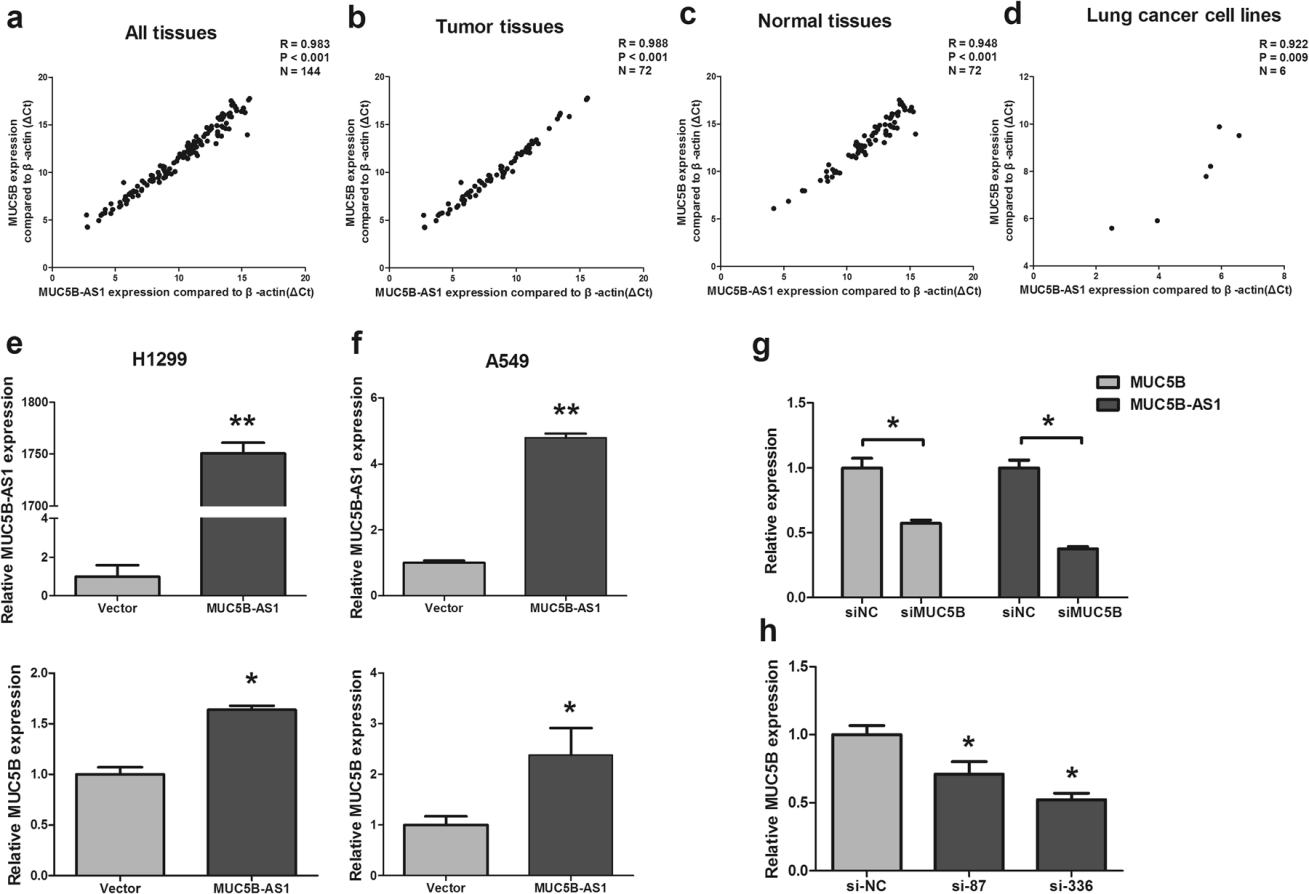
MUC5B-AS1 and MUC5B are co-expressed and mutually regulated in lung adenocarcinoma. **a** Correlation between MUC5B-AS1 and MUC5B mRNA expression in 72 paired tissues. **b**, **c** Correlation between MUC5B-AS1 and MUC5B mRNA expression in 72 lung tumor tissues and normal lung tissues. **d** Correlation between MUC5B-AS1 and MUC5B mRNA expression in six cell lines (A549, H1299, SPCA1, H1975, H460, and HBE). Correlation coefficient *R* and *P-*values were calculated by a Pearson correlation analysis. **e**, **f** MUC5B mRNA expression levels in MUC5B-AS1 overexpressing H1299 and A549 cells. Upper graph: MUC5B-AS1 expression levels. Lower graph: MUC5B mRNA expression levels. MUC5B-AS1 vs. control vector, Student’s *t*-test, *P < 0.05. MUC5B-AS1 vs. control vector, Student’s *t*-test, **P < 0.01. **g** MUC5B-AS1 expression levels were detected in A549 cells after knockdown of MUC5B mRNA by siRNA. si-MUC5B vs. si-NC, Student’s *t*-test, *P < 0.05. **h** MUC5B mRNA expression levels were detected in A549 cells after knockdown of MUC5B-AS1 by siRNA (si-87 and si-336). siRNA vs. si-NC, Student’s *t*-test, *P < 0.05

We next explored the relationship between MUC5B-AS1 and MUC5B gene. We detected MUC5B mRNA expression levels in the same 72 paired tissues as above using qRT-PCR. We found that MUC5B-AS1 expression levels were highly positively correlated with MUC5B mRNA (*R*^2^ = 0.983, *P* < 0.001, Fig. 5a). A subgroup analysis by tissue types revealed that MUC5B-AS1 were highly correlated with MUC5B mRNA both in lung tumor tissues (*R*^2^ = 0.988, *P* < 0.001, Fig. 5b) and adjacent normal lung tissues (*R*^2^ = 0.948, *P* < 0.001, Fig. 5c). MUC5B-AS1 also showed a high correlation with MUC5B mRNA in the six cell lines (A549, H1299, SPCA1, H1975, H460, and HBE) (*R*^2^ = 0.922, *P* < 0.001, Fig. 5d).

To identify whether MUC5B-AS1 was directly associate with MUC5B expression, we first investigated MUC5B expression levels in the above-mentioned gain-of-function cell models. Overexpression of MUC5B-AS1 in H1299 and A549 cells showed an increased level of MUC5B mRNA (*P* < 0.05) (Fig. 5e, f). The immunofluorescence analysis showed that overexpression of MUC5B-AS1 also increased MUC5B protein levels in H1299 and A549 cells (Supplementary Fig. S3). Furthermore, siRNA target sites were designed to target only non-overlapping (non-OL) regions of MUC5B mRNA (Supplementary Fig. S1e). MUC5B mRNA knockdown using non-OL siRNA also significantly reduced MUC5B-AS1 expression levels in A549 cells (*P* < 0.05) (Fig. 5g). In addition, MUC5B mRNA were also significantly downregulated upon knockdown of MUC5B-AS1 in A549 cells (Fig. 5h). These results indicated that the expression of MUC5B-AS1 and MUC5B influenced each other.

### MUC5B-AS1 increases the stability of MUC5B mRNA by forming a protective RNA duplex

Due to the fact that MUC5B-AS1 as a lncRNA whose expression is highly correlated with that of MUC5B, thus, we explored whether MUC5B and MUC5B-AS1 had identical regulatory elements. The UCSC genome browser displayed that MUC5B and MUC5B-AS1 have different promoters (Supplementary Fig. S4a). We further explored regulatory elements from the ORegAnno (Open Regulatory Annotation) track in the UCSC genome browser, which found 11 transcription factor binding sites (TFAP2C, FOXA1, CEBPA, HNF4A, CTCF, and ESR1) in MUC5B promoter region and 2 transcription factor binding sites (MAFF and MAFK) in MUC5B-AS1 promoter region (Supplementary Fig. S4a). MUC5B and MUC5B-AS1 had no common transcription factors in the promoter region.

**Fig. 6:**
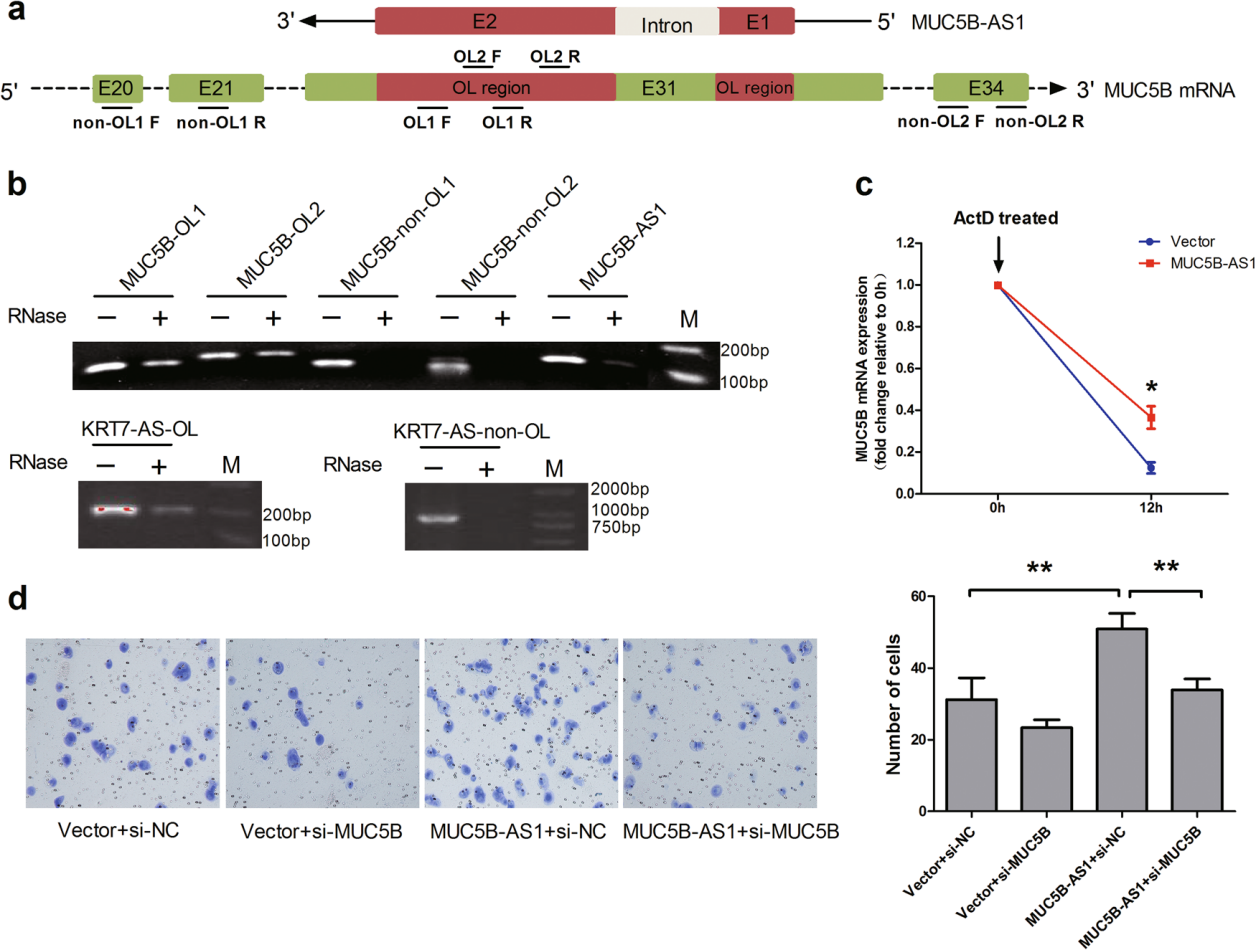
MUC5B-AS1 increases the stability of MUC5B mRNA by forming a protective RNA duplex. **a** Schematic representation of the PCR amplification regions for overlapping (OL) and non-overlapping (non-OL) regions of MUC5B. We designed two pairs of primers to amplify the OL regions (OL1 and OL2) and non-OL (non-OL1 and non-OL2) regions of MUC5B, respectively. F forward primer, R reverse primer. **b** RT-PCR products of OL and non-OL regions of MUC5B. Total RNA samples were treated with RNAse A + T cocktail and then cleaned up RNA using RNeasy kits. RT-PCR was conducted using the primers to detect the OL and non-OL regions of the MUC5B mRNA. OL and non-OL regions of KRT7-AS were used as a positive control. **c** Stability of MUC5B mRNA over 12 h was measured by qRT-PCR relative to time 0 h after blocking new RNA synthesis with Actinomycin D (1 μg/mL; indicated with black arrow). H1299 cells with MUC5B-AS1 or empty vector stable expression were treated with 1 μg/mL ActD, and then harvested cells for RNA purification at 12 h after addition of ActD. Then, MUC5B mRNA stability were subsequently measured by qRT-PCR and were normalized against a synthesized exogenous reference λ polyA^+^ RNA. Student’s *t*-test, *P < 0.05. **d** Rescue effect on migration by MUC5B siRNAs in H1299 cells with MUC5B-AS1 stable expression. Student’s *t*-test, **P < 0.01

The properties of nucleotide base complementary relationship between MUC5B-AS1 and MUC5B urged us to hypothesize that MUC5B-AS1 and MUC5B mRNA could form a protective duplex. We designed two pairs of primers to amplify the OL regions or non-OL regions of MUC5B, respectively (Fig. 6a). Ribonuclease protection assay (RPA) assay were used to test our hypothesis. The primers for amplifying the OL and non-OL regions of KRT7-AS (a known antisense lncRNA) were used as a positive control^25^. RT-PCR results revealed that the OL1 and OL2 regions of MUC5B mRNA were partially protected from RNase degradation, whereas the non-OL1 and non-OL2 regions were totally digested (Fig. 6b). In order to validate the direct binding between MUC5B-AS1 and MUC5B mRNA, a pull-down of *in vitro* transcribed biotin-labeled MUC5B-AS1 and detection of the MUC5B mRNA were conducted. An irrelevant lncRNA SFTA1P (NR_027082.1) was used as a negative control. The pull-down of biotin-labeled MUC5B-AS1 in A549 cells was significantly enriched for MUC5B mRNA compared to the negative control (Supplementary Fig. S4b). To further test whether MUC5B-AS1 could enhance the stability of MUC5B mRNA, we used Actinomycin D (ActD, 1 μg/mL), an inhibitor of RNA polymerase II^26^, to block new RNA synthesis in H1299 cells over a 12-h period. Then, MUC5B mRNA levels were subsequently measured and were normalized against a synthesized exogenous reference λ polyA^+^ RNA. We found that overexpressing of MUC5B-AS1 in H1299 cells showed an increased stability of MUC5B mRNA compared with cells transfected with an empty vector (*P* < 0.01) (Fig. 6c). On the other hand, Actinomycin D treatment were also conducted in the presence of MUC5B siRNA. The stability of MUC5B-AS1 was decreased in A549 cells transfected with si-MUC5B relative to the control (Supplementary Fig. S4c).

### MUC5B siRNA restores the MUC5B-AS1 function

To determine whether MUC5B-AS1 exerted its function through MUC5B in lung cancer cells, rescue experiments were conducted. H1299 cells with MUC5B-AS1 or empty vector stable expression were transfected with MUC5B siRNAs. Transwell migration assays indicated that knockdown of MUC5B could partially rescue the effect of MUC5B-AS1 on the cell migration (Fig. 6d).

### The high expression of MUC5B is significantly associated with poor outcomes in patients with lung adenocarcinoma

**Fig. 7:**
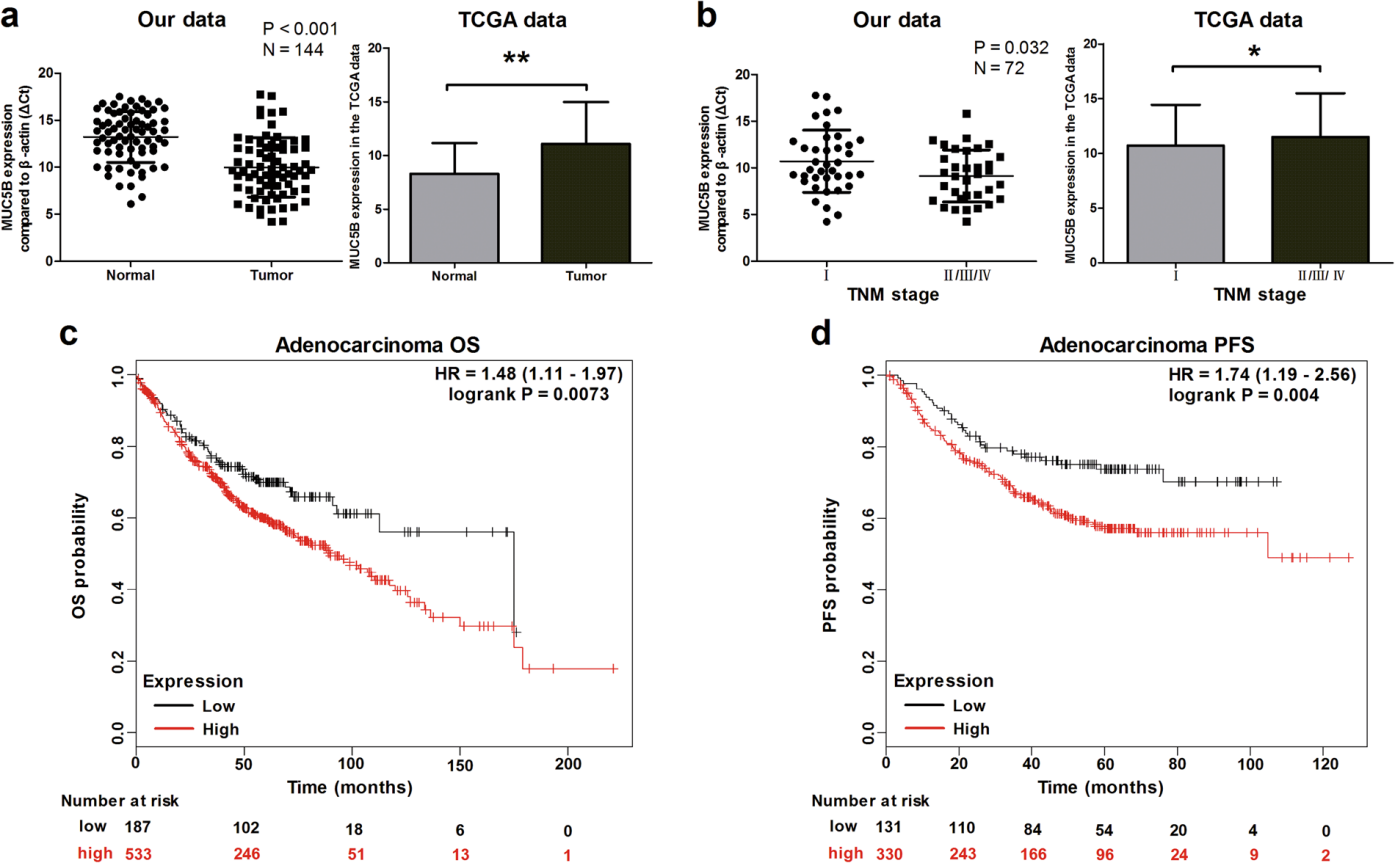
Expression patterns of MUC5B and its clinical significance. **a** Left: Expression analysis of MUC5B mRNA in lung adenocarcinoma tissues (*n* = 72) and paired adjacent normal lung tissues (*n* = 72). The ΔCt was used to show the expression level of MUC5B (ΔCt = Ct_MUC5B_–Ct_*β-actin*_). Lower ΔCt values indicate higher expression. Normal vs. tumor tissues, Student’s *t*-test. Right: MUC5B mRNA expression levels in the TCGA lung adenocarcinoma RNAseq (IlluminaHiSeq; *n* = 571) dataset (normal *n* = 58 vs. tumor *n* = 513). The results are expressed as the mean ± SD. Mann–Whitney U-test, **P < 0.001. **b** Left: The association of MUC5B mRNA expression with TNM stage in lung adenocarcinoma tissues (*n* = 72). Stage I vs. stage II/III/IV, Student’s *t*-test. Right: MUC5B mRNA expression levels were higher in patients with advanced TNM stage. Mann–Whitney U-test, *P < 0.05. **c** Kaplan–Meier curves for OS of lung adenocarcinoma patients (*n* = 720) with high and low expression levels of MUC5B mRNA. **d** Kaplan–Meier curves for PFS of lung adenocarcinoma patients (*n* = 461) with high and low expression levels of MUC5B

Since MUC5B-AS1 expression levels were highly positively correlated with MUC5B. We further explored MUC5B mRNA expression pattern using our qRT-PCR data and The Cancer Genome Atlas (TCGA) database. MUC5B mRNA levels were significantly upregulated in lung adenocarcinoma tissues compared with adjacent normal tissues (*P* < 0.001) (Fig. 7a). Consistent with our results, MUC5B mRNA expression levels were also significantly upregulated in TCGA lung adenocarcinoma tissues (*n* = 513) compared with normal lung tissues (*n* = 58) (*P* < 0.05) (Fig. 7a). MUC5B mRNA expression levels were also higher in stage II/III/IV patients than in stage I (*P* < 0.05) (Fig. 7b). We performed a meta-analysis to verify the association of MUC5B mRNA expression with outcomes among 1928 lung cancer patients using the Kaplan–Meier Plotter software program. We observed that high expression of MUC5B mRNA was significantly associated with poor OS (*n* = 720, hazard ratio (HR) = 1.48, 95% confidence interval (CI) = 1.11–1.97, *P* = 0.007) and progression-free survival (*n* = 461, HR = 1.74, 95% CI = 1.19–2.56, *P* = 0.004) in patients with lung adenocarcinoma (Fig. 7c, d). The association with OS remained significant by a multivariate Cox proportional hazards regression analysis after adjustment for gender, smoking, and tumor stage in adenocarcinoma (*n* = 387, HR = 1.89, 95% CI = 1.06–3.38, *P* = 0.031). However, the expression of MUC5B mRNA was not significantly associated with outcomes in lung squamous carcinoma (Supplementary Fig. S5).

## Discussion

In this study, we demonstrated that MUC5B-AS1, a new long non-coding antisense transcript for MUC5B, was significantly upregulated in lung adenocarcinoma tissues. MUC5B-AS1 promoted lung cancer cells migration and invasion *in vitro* and promoted metastasis *in vivo*. Furthermore, MUC5B-AS1 was co-expressed with MUC5B in lung, and increased the stability of MUC5B mRNA by forming a protective RNA duplex. Our results demonstrate that MUC5B-AS1 functions as an oncogenic lncRNA that promotes metastasis in lung adenocarcinoma by specifically regulating the MUC5B expression.

The lncRNAs can be categorized into five subcategories by their genomic location: intronic, intergenic, bidirectional, sense, and antisense lncRNAs^27^. Antisense lncRNAs are long non-coding RNAs that originate from opposite DNA strands of the same genomic locus^28,29^. The UCSC genome browser showed that MUC5B-AS1 was an antisense lncRNA that originate from the opposite strand of the MUC5B locus. As mentioned above, MUC5B, a member of the mucin family, has been implicated in the initiation or progression of several cancers including lung cancer. In the present study, we also evaluated the MUC5B mRNA expression patterns in lung tumor tissues. Consistent with previous research, MUC5B mRNA were also upregulated in lung tumor tissues compared with adjacent normal lung tissues and the expression levels of MUC5B mRNA were associated with TNM stage.

Several studies have indicated that sense/antisense expression patterns between tumor and normal samples could vary in a concordant (i.e., sense and antisense are both upregulated or both downregulated) or discordant (i.e., sense is upregulated, whereas antisense is downregulated or vice versa) manner^30,31^. Our data identified both MUC5B and MUC5B-AS1 were upregulated in a concordant fashion. Intriguingly, a highly positive correlation existed among the expressions of MUC5B/MUC5B-AS1 pair transcripts in both lung tumor tissues and normal lung tissues, and their high positive correlation was also observed in the lung cancer cell lines. We also explored that MUC5B-AS1 was directly associate with MUC5B mRNA expression by overexpressing MUC5B-AS1 or interfering MUC5B mRNA in lung cancer cell lines. The regulatory elements from the ORegAnno track in the UCSC genome browser suggested that MUC5B and MUC5B-AS1 may not be regulated by same transcription factors. Our results indicated that potential regulatory effects existed between MUC5B mRNA and MUC5B-AS1.

Antisense lncRNAs can be involved in the regulation of their neighboring gene at various levels:^31^ pre-transcriptionally (as protein guides^32^, or as decoys keeping proteins away from chromatin and as epigenetic regulators through histone modifications or DNA cytosine methylation^33–35^), transcriptionally (as modulators of transcription^36^), and post-transcriptionally (through RNA–RNA interactions that alter mRNA stability^37^). Antisense lncRNAs regulation at pre-transcriptional and transcriptional level operates mainly in the nucleus, while regulation at post-transcriptional level exerts either in the nucleus or the cytoplasm^5,31^. Our RNA FISH assays demonstrated that MUC5B-AS1 and MUC5B mRNA could be detected both in the nucleus and cytoplasm (mainly in the cell cytoplasm). The subcellular localization and nucleotide base complementary relationship indicate that MUC5B-AS1 may regulate sense gene MUC5B expression at post-transcriptional level by controlling mRNA stability through RNA–RNA interactions (double-stranded RNA). To test this hypothesis, we treated RNA samples with ribonuclease A + T, which digests single-strand RNAs but not RNA duplexes, and found that MUC5B-AS1 and MUC5B mRNA were capable of forming an RNA–RNA duplex at the complementary OL regions. RNA pull-down assays validated the physical interaction between MUC5B-AS1 and MUC5B mRNA. Moreover, overexpression of MUC5B-AS1 did enhance the stability of MUC5B mRNA under the treatment of Actinomycin D. These data suggested that MUC5B-AS1 increased MUC5B mRNA stability by forming an RNA–RNA duplex. Our results, in company with previous studies (BACE1-AS^37^, KRT7-AS^25^), demonstrated that antisense lncRNA may alter the secondary or tertiary structure of its sense mRNA by forming an RNA–RNA duplex, thus increasing its stability. In addition, another possible mechanism of regulation could be through translation and the impact of MUC5B-AS1 on MUC5B protein translation still needs further study.

We have demonstrated that MUC5B-AS1 could increase the cell migration and invasion abilities and that MUC5B-AS1 increased MUC5B mRNA stability, thereby one question was raised: whether MUC5B-AS1 promote cell migration by regulating MUC5B in lung cancer cells? Rescue experiments showed that MUC5B siRNA could partially restore the MUC5B-AS1 function. Our data suggest that MUC5B-AS1 acts as an oncogenic lncRNA that promotes cell migration by upregulating the expression of MUC5B in lung cancer. Previous studies have suggested that MUC5B could promote the cancer cell migration through the ERK1/2 signaling pathway^38^ or Wnt/β-catenin signaling pathway^39^. Based on the fact that MUC5B-AS1 expression levels were highly positively correlated with MUC5B mRNA and exerted its function through MUC5B in lung cancer cells, we further verified the prognostic significance of MUC5B mRNA. The high expression of MUC5B was associated with poor outcomes in lung adenocarcinoma. However, we did not find a significant association between MUC5B mRNA expression and outcomes in patients with lung squamous cell carcinoma.

In conclusion, this study demonstrates that MUC5B-AS1 is upregulated and functions as an oncogene in lung adenocarcinoma. MUC5B-AS1 promotes cell migration and invasion by forming a protective RNA–RNA duplex with MUC5B, thereby increasing MUC5B mRNA expression levels in lung cancer. MUC5B-AS1 cognate sense transcript MUC5B is also upregulated and the high expression of MUC5B is associated with poor outcomes in lung adenocarcinoma. These findings highlight the significance of MUC5B-AS1 upregulation in tumor metastasis and implicate MUC5B-AS1 as an attractive candidate target for lung adenocarcinoma treatment.

## Materials and methods

### Human tissue samples

Patients newly diagnosed with lung cancer were enrolled at Xinqiao Hospital of the Third Military Medical University in Chongqing, China. All tissues were collected prior to any radiation or chemotherapy during operation and stored at −80 °C prior to RNA extraction. The available fresh frozen lung tumors and matched normal lung tissues were sectioned and reviewed by a pathologist to confirm the diagnosis of lung cancer, tumor grade, histological cell type, tumor purity, and lack of tumor contamination in the normal lung. Tumor samples with ≥70% tumor-cell content and matched normal lung tissues from 72 cases were used in the study (Supplementary Table S1). Informed consent was obtained from all patients. Research protocol was approved by the ethics committee of the Third Military Medical University (Chongqing, China).

### Cell lines and animals

The lung cancer cell lines (A549, H1299, SPCA1, H1975, and H460) and human bronchial epithelium (HBE) cells were obtained from the Cell Bank of the Chinese Academy of Science (Shanghai, China) and the American Type Culture Collection (Manassas, VA, USA). Cells were cultured in RPMI-1640 (HyClone, Logan, UT, USA) or F12K (Gibco, Life Technology, Carlsbad, CA, USA) supplemented with 10% fetal bovine serum (HyClone, Logan, UT, USA). BALB/c-nude mice (4-week-old) were obtained from the Experimental Animal Center of the Chinese Academy of Science (Shanghai, China). Housing and all experimental animal procedures were approved by the Institutional Animal Care and Use Committee of Third Military Medical University.

### RNA extraction and qRT-PCR analysis

Total RNA was isolated using the TRIzol reagent (TaKaRa, Dalian, China). Real-time PCR was performed using the SYBR Premix Ex Taq (TaKaRa) following the manufacturer’s instructions. Results were normalized to the expression levels of *β-actin*. The ΔCt was used to show the gene expression levels and lower ΔCt values indicated higher expression. ΔCt = Ct_target_–Ct_*β-actin*_. Primers sequences are shown in Supplementary Table S2.

### Plasmid construction and cell transfection

To construct a plasmid expressing MUC5B-AS1, the full-length human MUC5B-AS1 sequence (Ensembl: ENST00000532061.2) was synthesized and subcloned into the pcDNA3.1 vector (Sangon, Shanghai, China). The plasmids were transfected using Lipofectamine2000 Reagent (Invitrogen, NY, USA). The stably transfected cells were screened under G418 (Sangon). For knockdown of MUC5B mRNA, siRNA sequences (GenePharma, Shanghai, China) were transfected into cells with Lipofectamine 2000 (Invitrogen). MUC5B siRNA sequences are shown in Supplementary Table S2. For knockdown of MUC5B-AS1, we synthesized two independent single-strand siRNAs (single antisense strand) targeting MUC5B-AS1 to avoid the interference of MUC5B (Supplementary Table S2).

### CCK-8 assay, colony formation assay, cell migration, and invasion assays *in vitro*

Cell Counting Kit-8 (CCK-8) assay, colony formation assay, cell migration, and invasion assays were performed as described previously^40^.

### Immunofluorescence cell staining

Cells were grown on sterile glass coverslips, fixed in 4% paraformaldehyde for 15 min, permeabilized by 0.1% Triton X-100, and then blocked with 1% BSA for 30 min. Mouse anti-MUC5B (Abcam Inc., Cambridge, MA) were incubated overnight at 4 °C. After washing three times, the cells were probed with Alexa-Fluor-488-conjugated donkey anti-mouse-IgG (Invitrogen) for 1 h at room temperature, followed by nuclear counterstaining with DAPI (Beyotime, China). Coverslips were observed with a fluorescence microscope (ZEISS, Germany).

### Lung metastasis model

Ten male BALB/c-nude mice (4-week-old) were randomly divided into two groups, with five mice in each group. H1299 cells with MUC5B-AS1 or empty vector stable expression were transfected with LV16 (U6/Luciferase17&Puro) vector (GenePharma, Shanghai, China) and were injected into the tail veins of the nude mice (1 × 10^6^ cells per mice). Optical *in vivo* imaging of cancer metastasis was monitored with *in vivo* luminescence imaging system (IVIS) at 3, 4, and 5 weeks post injection. The mice were sacrificed 5 weeks after injection and the lungs were removed for further analysis. To further confirm the pro-metastatic potential of MUC5B-AS1, A549 cells (1 × 10^6^) stably transfected with MUC5B-AS1 or control vector were injected into the tail veins of nude mice. The mice were sacrificed 4 weeks after injection and the lungs were removed for further analysis.

### RNA fluorescence in situ hybridization

Cy3-labeled MUC5B-AS1 or MUC5B mRNA probes were obtained from RiboBio (Guangzhou, China). RNA FISH were performed as described previously^41^ using fluorescent in situ hybridization kit (RiboBio) following the manufacturer’s instructions. U6 snRNA and 18S rRNA probes (RiboBio) were used as positive control.

### Ribonuclease protection assay

We designed two pairs of primers to amplify the OL regions or non-OL regions of MUC5B, respectively. Primer sequences are listed in Supplementary Table S2. RPA assay was performed as Huang described^25^. Briefly, RNA samples were incubated at 37 °C for 1 h and then treated with RNAse A + T cocktail (AM2286, Thermo Fisher Scientific, Waltham, MA, USA) for 30 min at 37 °C. Subsequently, the samples were incubated with proteinase K (Thermo Fisher Scientific) for 1 h at 50 °C. We then cleaned up RNA using RNeasy kits (QIAGEN, Madison, WI, USA) and carried out reverse transcription-polymerase chain reaction (RT-PCR) using the primers to detect the OL and non-OL regions of the MUC5B mRNA. The primers sequences are shown in Supplementary Table S2.

### RNA pull-down

MUC5B-AS1 and lncRNA-SFTA1P (negative control) were *in vitro* transcribed, respectively, from vector pcDNA3.1-MUC5B-AS1 and pcDNA3.1-SFTA1P, and biotin-labeled with the Biotin RNA Labeling Mix (Roche, Mannheim, Germany) and T7 RNA polymerase (Roche), treated with RNase-free DNase I (Promega, Madison, WI, USA). The biotin-labeled RNA were purified with an RNeasy Mini Kit (Qiagen). Cell lysates from A549 cells (1 × 10^7^) were incubated with 3 μg of purified biotin-labeled transcripts for 1 h at 25 °C. The biotin-labeled RNA were isolated with Dynabeads^TM^ M-270 Streptavidin (Invitrogen). The MUC5B mRNA present in the pull-down complexes were detected by qRT-PCR analysis.

### RNA stability and Actinomycin D treatment

H1299 cells with MUC5B-AS1 or empty vector stable expression were seeded onto 6-well plates. We treated these cells with Actinomycin D (ActD, 1 μg/mL), and then harvested cells for RNA purification at 12 h after addition of ActD. MUC5B mRNA levels were subsequently measured by qRT-PCR and were normalized against a synthesized exogenous reference λ polyA^+^ RNA (Takara, China) following the manufacturer’s instructions^42^. To test whether MUC5B can regulate the stability of MUC5B-AS1, A549 cells were seeded in a 6-well plate and transfected with si-MUC5B or si-NC (negative control siRNA). We treated these cells with ActD (1 μg/mL) at 6 h post transfection. MUC5B-AS1 expression levels were subsequently measured by qRT-PCR at 12 h after addition of ActD.

### Bioinformatics analysis

The TCGA lung adenocarcinoma RNAseq (IlluminaHiSeq, *n* = 571) data were used to analyze the expression patterns of MUC5B mRNA in the TCGA lung adenocarcinoma. To analyze the prognostic value of MUC5B, Kaplan–Meier curves were generated using the Kaplan–Meier Plotter (KMplot) program (www.kmplot.com)^43^. The gene expression data and relapse-free and OS information in the KMplot program are downloaded from Gene Expression Omnibus, European Genome-phenome Archive and TCGA datasets. The patients were split into two groups according to the auto select best cutoff of MUC5B probe (213432_at). For a multivariate Cox proportional-hazards regression analysis, we adjusted for gender, smoking status, and tumor stage.

### Statistical analysis

The results were expressed as the mean ± SD. Results of gene expression, cell growth, migration, and invasion were evaluated using the two-tailed Student’s *t*-test. Pearson correlation was to analyze the correlation between MUC5B-AS1 and MUC5B mRNA expressions. A two-sided *P*-value less than 0.05 was taken as statistically significant. Statistical analyses were performed with the SPSS 22.0 software (SPSS, Inc., Chicago, IL, USA).

## Electronic supplementary material

Suplementary Figures and Tables
