# Supplementary material for: Long non-coding RNA MUC5B-AS1 promotes metastasis through mutually regulating MUC5B expression in lung adenocarcinoma
Source: Cell Death Dis. 2018 Apr 18;9(5):450. doi: 10.1038/s41419-018-0472-6 (PMC5906460; doi:10.1038/s41419-018-0472-6)
Supplement: Supplementary file 1 — Suplementary Figures and Tables [file 41419_2018_472_MOESM1_ESM.doc]

**Supplementary Table S1.** Clinical characteristics of 72 lung adenocarcinoma patients for qRT-PCR analyses

| **Patient characteristics** | **Adenocarcinoma**  **n=72** |  |
| --- | --- | --- |
| **Age at diagnosis (years), Mean (SD)** | 55.93 (12.38) | |
| **Gender (%)** |  | |
| Male | 35 (48.6) | |
| Female | 37 (51.4) | |
| **Tumor stage (%)** |  | |
| I | 38 (52.8) | |
| II | 21 (29.2) | |
| III | 10 (13.9) | |
| IV | 3 (4.2) | |
| **Lymph nodes metastasis** |  | |
| Absent | 48 (66.7) | |
| Present | 24 (33.7) | |
| **Smoking history (%)** |  | |
| Never-smoker | 34 (47.2) | |
| Smoker | 30 (41.7) | |
| Not available | 8 (11.1) | |

**Supplementary Table S2.** Primers and RNA oligonucleotides sequences used in this study.

| **Gene** | **Sequence (5’-3’)** | **Length (bp)** |
| --- | --- | --- |
| **MUC5B** | Forward: GTGAGGAGGACTCCTGTCAAGT | 90 |
|  | Reverse: CCTCGCAGAAGGTGATGTTG |  |
| **MUC5B-AS1** | Forward: CTCTGTGAGGATCCAGTGGACG | 150 |
|  | Reverse: TGTGCTTTGCTGTGACGACT |  |
| ***β-actin*** | Forward: CCACGAAACTACCTTCAACTCC | 132 |
|  | Reverse: GTGATCTCCTTCTGCATCCTGT |  |
| **MUC5B-OL1** | Forward: TGACGTGGACTTCCCAACCT | 146 |
|  | Reverse: CCTGGTCGATGCTTACCTCG |  |
| **MUC5B-OL2** | Forward: TGGGCACCAAAGAGCATAGAG | 177 |
|  | Reverse: GTCGTCACAGCAAAGCACAC |  |
| **MUC5B-non-OL1** | Forward: GGCTGTTTCAGCACACACTG | 123 |
|  | Reverse: CTTGTAGGTGGCCTCGTTGT |  |
| **MUC5B-non-OL2** | Forward: TTGACGGCACCTCTTACACC | 101 |
|  | Reverse: GTGGTTGTCCAGGTAGAGGC |  |
| **KRT7-AS-OL** | Forward: GTAGGTGGCGATCTCGATGTC | 171 |
|  | Reverse: ATTGCCGAGGCTGAGGAG |  |
| **KRT7-AS-non-OL** | Forward: GGCCTGAATGTCCAAGGCT | 902 |
|  | Reverse: ACGTAGGACATGTTTCTGCCC |  |
| **MUC5B siRNA** | Sense: GTCTGCAGGAACCGTGAGCAG |  |
| **si-87** | Single strand: ACCUGGAUCCUACAAAGC |  |
| **si-336** | Single strand: AUGUGCUGGGCACCAAAGA |  |


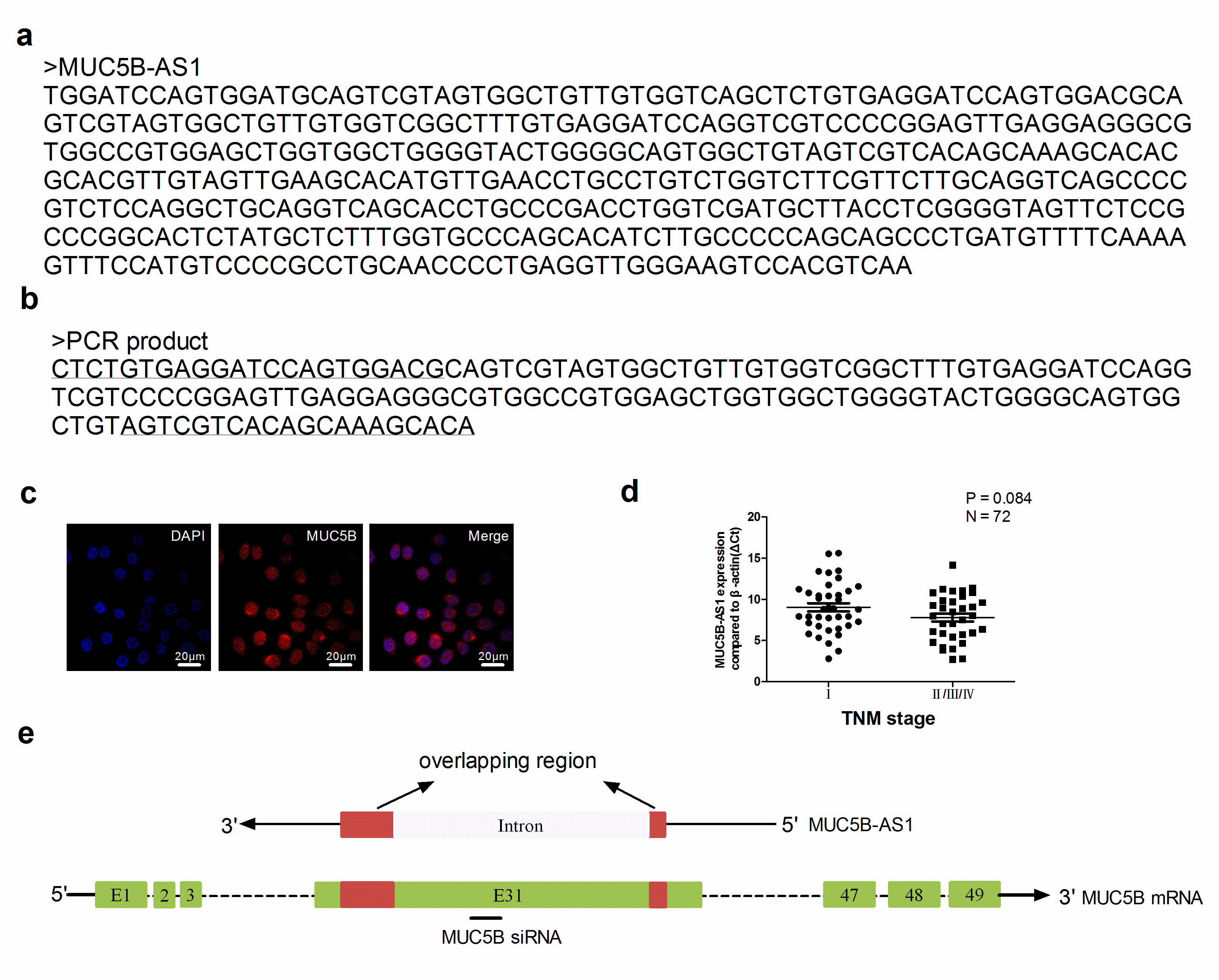


**Supplementary Figure S1.** (a) The full sequence of MUC5B-AS1 (434 nt). (b) PCR product was confirmed by DNA sequencing. The sequencing results showed that the primer of MUC5B-AS1 avoid the non-specific amplification of MUC5B mRNA or genomic DNA. (c) Localization of MUC5B mRNA by RNA-FISH. Blue, DAPI-stained nuclei; red, Cy3-labeled positive hybridization signals (scale bar, 20 μm). (d) The association of MUC5B-AS1 expression with TNM stage in lung adenocarcinoma tissues (n = 72). Stage I vs. stage II/III/IV, Student’s t-test. The ΔCt was used to show the expression level of MUC5B-AS1 (ΔCt = Ct_MUC5B-AS1_ – Ct*_β-actin_*). Lower ΔCt values indicate higher expression. (e) Schematic representation of the target site of MUC5B siRNA. The schema is not drawn to scale. The siRNA was designed to target only non-OL regions of MUC5B mRNA.


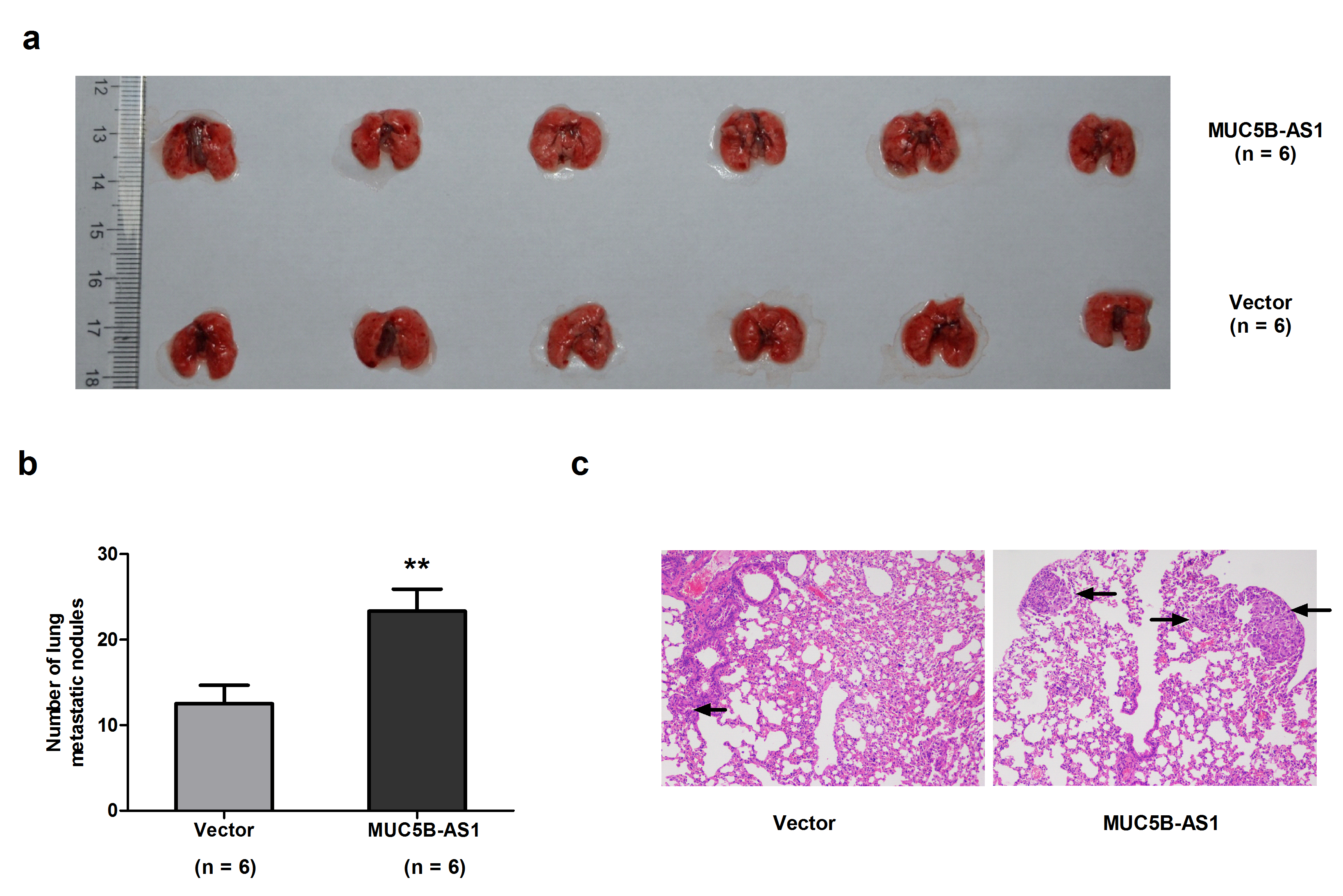


**Supplementary Figure S2.** Overexpression of MUC5B-AS1 promotes A549 cells metastasis *in vivo*. (a) Photograph of entire lungs from nude mice in each group 4 weeks after injections of A549 cells. (b) The number of lung metastatic nodules on lung surfaces were counted. ** MUC5B-AS1 vs. control vector, Student’s t-test, P < 0.01. (c) Representative H&E staining of lung tissue slices confirmed that more metastatic nodules were present in MUC5B-AS1 group than vector control group (100×)


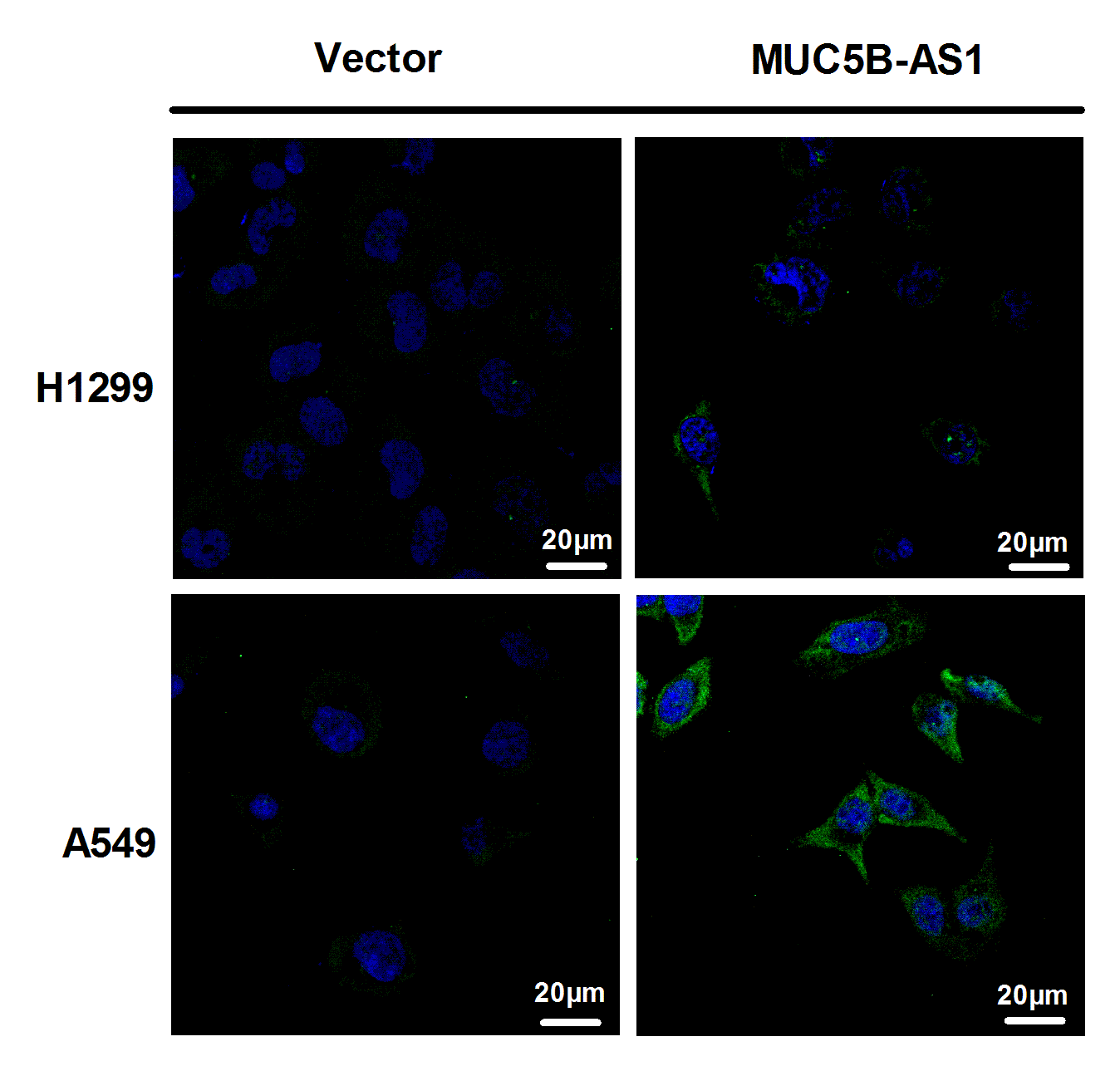


**Supplementary Figure S3.** MUC5B protein expression was examined by immunofluorescence analysis. MUC5B protein expression was up-regulated in H1299 and A549 cells with MUC5B-AS1 overexpression compared with vector control. Blue, DAPI-stained nuclei; green, MUC5B. Scale bar: 20 μm.


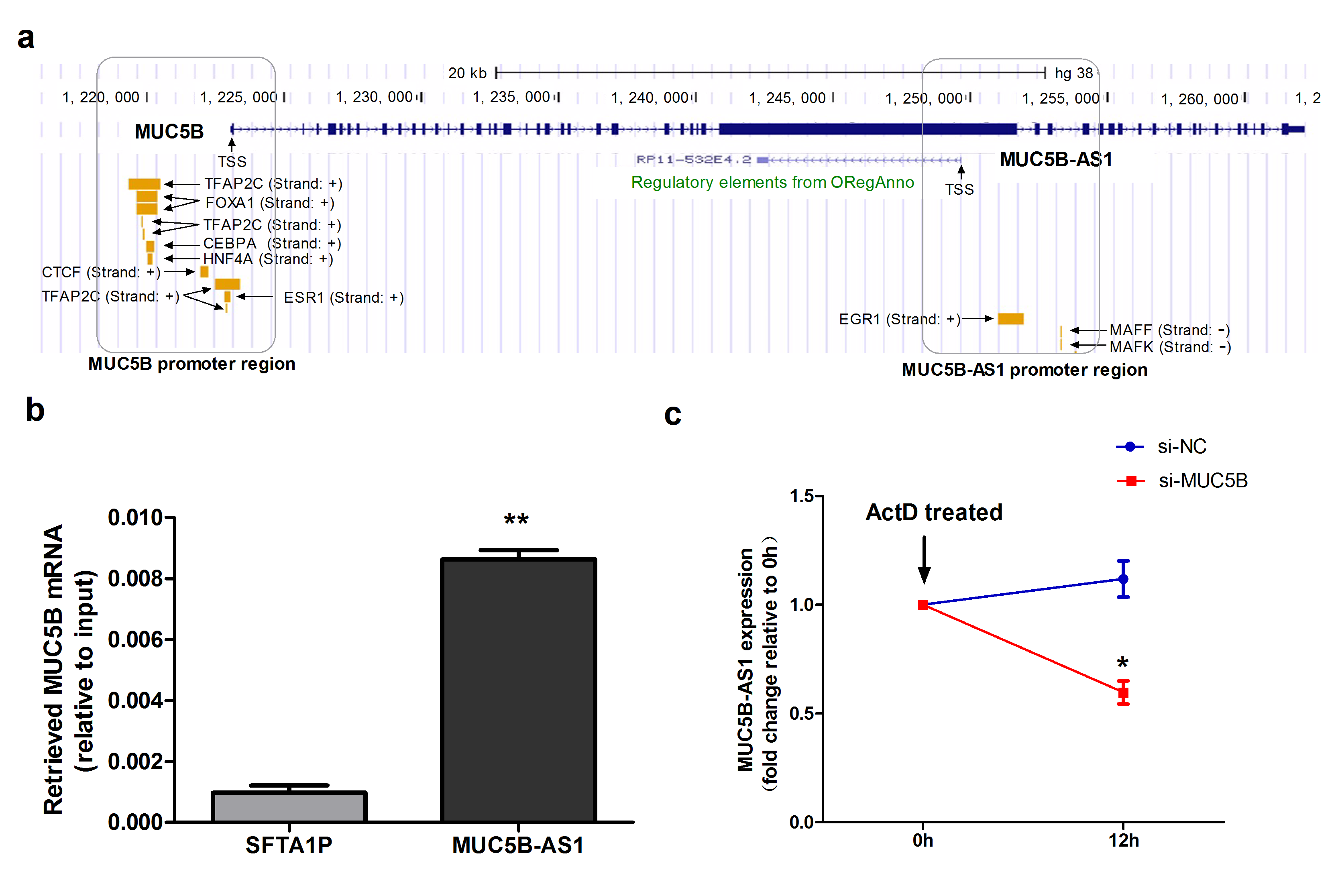


**Supplementary Figure S4.** (a) Potential transcription factor binding sites in MUC5B and MUC5B-AS1 promoter regions. The transcription factor binding sites were shown in orange. TSS, transcriptional start site. (b) A549 cell lysates were incubated with biotin-labeled MUC5B-AS1 or SFTA1P. After pull-down, RNA was extracted and assessed by qRT-PCR. ** MUC5B-AS1 vs. SFTA1P, P < 0.01. (c) Stability of MUC5B-AS1 over 12h was measured by qRT-PCR relative to time 0h after blocking new RNA synthesis with Actinomycin D (1μg/mL; indicated with black arrow). A549 cells transfected with si-MUC5B or si-NC were treated with 1μg/mL ActD, and then harvested cells for RNA purification at 12h after addition of ActD. Then, MUC5B-AS1 stability were subsequently measured by qRT-PCR and were normalized against a synthesized exogenous reference λ polyA^+^ RNA. *Student’s t-test, *P* < 0.05.


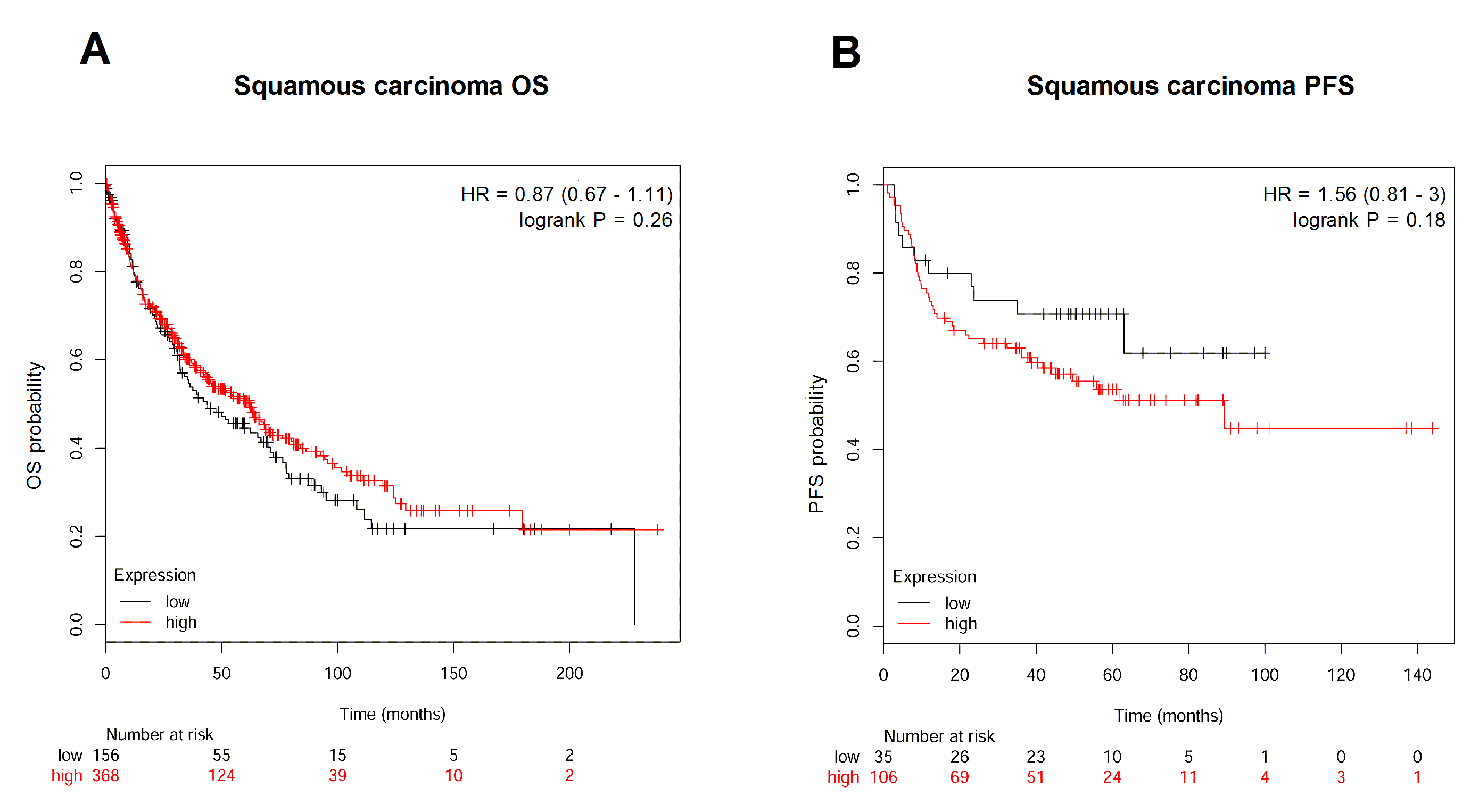


**Supplementary Figure S5.** MUC5B mRNA was not associated with outcomes in squamous carcinoma. (A) Kaplan-Meier curves for overall survival (OS) of lung squamous carcinoma patients (n = 524) expressing high and low expression levels of MUC5B mRNA. (B) Kaplan-Meier curves for progression free survival (PFS) of lung adenocarcinoma patients (n = 141) expressing high and low expression levels of MUC5B.
